# Feasibility of Tablet Computer Screening for Opioid Abuse in the Emergency Department

**DOI:** 10.5811/westjem.2014.11.23316

**Published:** 2014-12-17

**Authors:** Scott G. Weiner, Laura C. Horton, Traci C. Green, Stephen F. Butler

**Affiliations:** * Brigham and Women’s Hospital, Department of Emergency Medicine, Boston, Massachusetts; † Tufts University School of Medicine, Boston, Massachusetts; ‡ Rhode Island Hospital, Department of Emergency Medicine, Providence, Rhode Island; § Inflexxion, Inc. Newton, Massachusetts

## Abstract

**Introduction:**

Tablet computer-based screening may have the potential for detecting patients at risk for opioid abuse in the emergency department (ED). Study objectives were a) to determine if the revised Screener and Opioid Assessment for Patients with Pain (SOAPP®-R), a 24-question previously paper-based screening tool for opioid abuse potential, could be administered on a tablet computer to an ED patient population; b) to demonstrate that >90% of patients can complete the electronic screener without assistance in <5 minutes and; c) to determine patient ease of use with screening on a tablet computer.

**Methods:**

This was a cross-sectional convenience sample study of patients seen in an urban academic ED. SOAPP®-R was programmed on a tablet computer by study investigators. Inclusion criteria were patients ages ≥18 years who were being considered for discharge with a prescription for an opioid analgesic. Exclusion criteria included inability to understand English or physical disability preventing use of the tablet.

**Results:**

93 patients were approached for inclusion and 82 (88%) provided consent. Fifty-two percent (n=43) of subjects were male; 46% (n=38) of subjects were between 18–35 years, and 54% (n=44) were >35 years. One hundred percent of subjects completed the screener. Median time to completion was 148 (interquartile range 117.5–184.3) seconds, and 95% (n=78) completed in <5 minutes. 93% (n=76) rated ease of completion as very easy.

**Conclusions:**

It is feasible to administer a screening tool to a cohort of ED patients on a tablet computer. The screener administration time is minimal and patient ease of use with this modality is high.

## INTRODUCTION

Screening tools to detect undiagnosed mental health and substance use problems have been developed to enable earlier detection of disorders, and thus, earlier care.^1^ Multiple tools have been developed for this purpose, including the Patient Health Questionnaire (PHQ-9) for depression, Alcohol Use Disorders Identification Test (AUDIT) and the Drug Abuse Screening Test (DAST).^2^–^4^ These tools are an important first step in the process of SBIRT (screening, brief intervention and referral to treatment).^5^ Using such screening tools in the emergency department (ED) can be powerful, particularly at the time of exacerbation of disease.^6^,^7^

The process of screening patients may be time consuming, costly and can require staff resources that do not exist.^8^ Computerized screening may be a solution to this dilemma.^9^ Computerized screening requires minimal staff time, scores are calculated without error and, with the recent increased number of available products and expanded use of tablet computers in society over the past several years, patients are becoming comfortable interacting with technology. Given these factors and the evolution of tablet computers that are now lighter, less expensive and with a longer battery life,^10^ screening ED patients with tablet computers may be an attractive option.

In this study, we used an electronic tablet version of a screener for opioid prescription abuse potential. Opioid prescription abuse in the United States has increased exponentially over the past decade.^11^ Deaths from drug overdose have surpassed deaths from motor vehicle accidents, and the problem has been described as an epidemic,^12^,^13^ elevating screening for opioid abuse potential to great importance.

The screening tool we chose for our ED population is the Revised Screener and Opioid Assessment for Patients with Pain (SOAPP®-R).^14^ This proprietary screening measure, developed and validated by Inflexxion, Inc. as part of a NIDA-funded Small Business Innovative Research (SBIR) grant, was developed and validated in pain clinic patients and is also commonly used in primary care practices. The Centers for Disease Control and Prevention have concluded: “Health-care providers should only use opioid pain relievers in carefully screened and monitored patients when non-opioid pain reliever treatments are insufficient to manage pain.”^11^ Despite the fact that up to 42% of ED visits are for painful conditions^15^ and that emergency physicians commonly prescribe opioids, screening tools like this are not commonly used in the ED setting.

Our study has the following objectives: a) To determine if this screening tool could be administered on a tablet computer in an ED patient population; b) To demonstrate that >90% of patients can complete the screener without assistance in <5 minutes and; c) To determine patient perception of ease of use with screening on a tablet computer.

## MATERIAL AND METHODS

### Study Location

This was a cross-sectional, prospective, convenience sample study of patients seen at a single urban academic Level I trauma center with approximately 42,000 annual visits. The protocol was approved as exempt by our hospital’s institutional review board. Patient consent was determined by the patient indicating willingness to continue on the welcome screen of the tablet computer program.

### Programming the Tablet Screener

SOAPP®-R was administered on a generic seven-inch tablet running the Android operating system (PC709 Android 4.0 Tablet, dimensions 7×5×0.25 inches). Permission to use the SOAPP®-R instrument in electronic format for this study was granted by its copyright holder (Inflexxion, Inc., Newton, MA). The tablet was programmed using the “App Inventor” programming language.^16^ In addition to the screening tool, basic demographic questions and a final question asking satisfaction/ease of use with the tablet screener were included.

### Patients

Included individuals were patients ages ≥18 years who were being considered for discharge with an opioid analgesic by the attending emergency physician. Exclusion criteria were the following: inability to understand English, physical disability preventing use of the tablet, the patient was not being prescribed an opioid for the treatment of acute or chronic pain (e.g. codeine given for cough suppression or buprenorphine or methadone for maintenance of a drug treatment program), dementia or other mental impairment, or the patient was a prisoner.

### Intervention

Patients were identified by physicians informing the research assistant that they were being discharged with an opioid analgesic, or when the research assistant saw on the electronic charting system (Medhost EDIS, Medhost, Inc., Plano TX) that the patient was being discharged with such a prescription. This trained researcher approached the patient, briefly described the study, and handed them the tablet with the survey program open. Consent was acknowledged on the tablet, and a welcome screen informed patients that their responses would not be shared with their treating clinicians, and thus, not affect medications prescribed to them. Although the researcher was present at all times, patients were required to complete the screener without assistance. The researcher was also unaware of the patients’ screening results, which were stored only on the tablet for later analysis and not reported at the time of screening. The internet functionality of the tablet was disabled to prevent possible breach of data, and the tablet was stored in a locked safe at the clinical site when not in use. Data were exported to a computer in a locked office on a weekly basis during the study.

The SOAPP®-R is a 24-question screening tool that has a question stem followed by one of five responses, each with an associated number of points: never (0 points), seldom (1 point), sometimes (2 points), often (3 points), and very often (4 points). Therefore, the range of total points possible is 0–96. A positive score on the screener, which has been identified as predicting aberrant medication-related behavior within six months after initial testing, is 18 points or higher. This score was determined to have a sensitivity of 81% for detecting high-risk patients.^14^ The tool was originally designed to be administered on paper and completed in less than 10 minutes (600 seconds). A screen shot of the tablet version is found in the Figure.

### Outcome Measures

The three outcome measures were a) to determine if the SOAPP®-R could be administered on a tablet computer to an ED patient population, determined by survey completion rate; b) to demonstrate that the vast majority of patients can complete the electronic screener without assistance in <5 minutes (an arbitrary cutoff we thought would be most reasonable for patients and clinicians) and; c) to determine patient ease of use with screening on a tablet computer determined by a survey question built in to the tablet application asking patients to describe their experience as one of five choices: very easy, somewhat easy, neutral, somewhat difficult, or very difficult.

## THEORY/CALCULATION

### Power Calculation

Our sample size was based on calculations for a companion study comparing SOAPP-R scores with prescription drug monitoring data. We estimated that 30% (+/− 10%) of patients who completed SOAPP®-R would score as “at-risk” (score ≥18). The necessary sample size to obtain that margin of error with a 95% CI was determined to be 81 patients. This estimate was based on a prior study at our site showing that 33.1% of patients had evidence of aberrant drug-related behavior (≥4 opioid prescriptions and ≥4 providers in a 12-month period) on the state prescription drug monitoring program database.^17^ We purport that this number of patients is also sufficient for gathering adequate pilot data for this study.

### Statistical Analysis

We exported data from the tablet to a desktop computer and imported the data into statistical analysis software. There was no manual transfer of data required, so risk of data loss was negligible. Descriptive statistics were generated. We calculated mean, standard deviation, median, and minimum and maximum values for all continuous variables. Frequencies and percentages were calculated for all categorical variables. We analyzed all data with JMP v8.0 (SAS Institute, Inc., Cary, NC).

## RESULTS

### Patients

Between May and August 2013, 93 patients were approached for inclusion, and 82 (88%) provided consent. Patient characteristics are demonstrated in Table.

### Outcome Measures

One hundred percent of subjects were able to complete the tablet screener without assistance. Every patient completed the screener, answering all of the questions. Distribution of time to completion was not parametric. The median time to completion of the 24 questions on the SOAPP®-R was 148.0 seconds (interquartile range=117.5–185.3). Seventy-eight of 82 patients (95.1%) were able to complete the screener in <300 seconds (5 minutes). The mean SOAPP®-R score was 16.0 (95% CI 13.2–18.8). Approximately one third (32.9%, n=27) of patients had a SOAPP®-R score ≥18, indicating that they were “at risk” for aberrant behavior.

Patients rated ease of completion as 93% (n=76) very easy, 1% (n=1) somewhat easy, 5% (n=4) neutral, 1% (n=1) somewhat difficult. Overall, the tablet had no malfunctions and operated normally throughout the study.

## DISCUSSION

This study demonstrated that a screening tool for opioid abuse potential can be administered electronically to an ED patient population. Our research joins multiple prior studies in various clinical settings demonstrating the applicability and feasibility of electronic screening. Early studies of computerized screening in healthcare settings were performed before the introduction of tablet computers, and focused mainly on the fidelity between paper and electronic versions of the screener. For example, Olajos-Clow et al. studied patients completing the Mini Asthma Quality of Life questionnaire.^18^ Patients were randomized to either a paper or a computerized version. The researchers found that there was good agreement between the two methods and that the electronic version was preferred by most participants. Similar findings were present in other crossover comparison studies of electronic versus original paper versions.^19^–^22^

Other studies have looked at technology-based screening specifically in the ED patient population. Cotter et al. surveyed adolescents and young adults about their energy drink and caffeinated beverage use, administered on a tablet computer.^23^ Ewing et al. administered the computerized alcohol screening and intervention (CASI) system to screen over 1,000 traumatized patients for alcohol use with the aforementioned AUDIT tool in electronic format.^24^ And although not for screening purposes, an interactive computerized history-taking program has been successfully used to augment history information at triage without delaying patient care.^25^

In a large study, Ranney et al. interviewed 664 ED patients about their use of technology.^26^ The study found that baseline use of computers and mobile phones was high (>90%) in their patient population, although the methodology oversampled adolescents/young adults, and mean patient age was 31 years. Patients were concerned about their confidentiality in regards to the internet and social media, but were interested in technology-based behavioral health interventions.

All of these studies confirm that patients can interact with the technology. That said, one of our concerns at the onset of this research was truthfulness of patients. It would be easy to simply select the same answer for each question or not answer honestly. One of the earliest studies to evaluate this problem was Lucas et al. in 1977.^27^ Using a primitive computer system, it was determined that patients being screened for alcohol consumption reported significantly greater amounts of alcohol use to the computer than they reported to psychiatrists asking the same question. Our results, demonstrating that 32.9% of patients had a score of 18 points or higher (“at-risk”) on the SOAPP®-R screener, suggest they were most likely being truthful and is remarkably consistent with our prior research indicating that 33.1% of patients with back pain, headache or dental pain exhibited aberrant medication use behavior.^17^ It must be emphasized that patients were told that the results were not going to be shared with their treating clinician. If they had been, results may have varied. Future dedicated research on the accuracy of the screener must be done before any conclusions can be made about this aspect of the screening tool. Furthermore, it is not known what steps emergency clinicians would take after they learn about a positive screening result for one of their patients.

There are also studies describing the downsides of such technology. For example, while initial reports of diagnostic computer kiosks were positive, Ackerman and colleagues described the failure of kiosks in their EDs and concluded that there are context-related factors involved in implementation of information technology projects into complex medical settings.^28^ The study serves as a warning that what is feasible in one hospital may not work in others.

There are important factors to consider with self-programming of a tablet screener, such as a possible copyright infringement if permission to use commercial screener is not obtained, issues of collection and protection of protected health information (especially when dealing with sensitive issues such as substance abuse histories and other highly confidential patient data), and eventual integration into an electronic medical record. The developers of the SOAPP®-R at Inflexxion do offer a commercially available tablet version (the Pain Assessment Interview Network—Clinical Advisory System – “PainCAS”).

This study supports three concepts. The first is that, with graphics-based programming languages like App Inventor, it is now possible for clinicians with minimal prior programming experience to create programs that can be used in the clinical setting, rendering development and implementation costs minimal. The second is that patients are able to interact with the technology of tablet computers in the ED setting, find them easy to use and appear to respond truthfully to the questions asked on a screener. The third concept is that, because it is electronic, there is little chance of data loss and exact times to completion of the survey can be recorded. Our app recorded the exact time taken from the first question of SOAPP®-R appearing on the screen to answering the last question, allowing for a precise measurement of time that did not rely on a researcher.

## LIMITATIONS

As this was a convenience sample, selection bias may have been present. The study was conducted when research staff was available to enroll so only a small percentage of potentially eligible subjects was enrolled. We only included patients who were fluent in English and might have therefore excluded at-risk minority populations. Furthermore, because this is a single center study in an urban environment, the results may not be externally applicable to other patient populations. Specifically, we do not know if our patient population has more experience using tablet computers than others. Only 6.1% of our patients were aged 56 or older, so it is not possible to comment on the use of the tablet computer in the elderly population. Although about one-third of patients had an “at-risk” SOAPP®-R score, it is possible that patients were not truthful with the results. Alternatively, because patients knew that the results would not be reported to their treating clinician, they may have been honest when they would not have been if they feared that their answers would prevent them from receiving an opioid pain reliever.

Configuration of the tablet response buttons (vertical layout) is different than the paper version (horizontal layout) and may have predisposed patients towards simply the top answers (i.e. never or seldom), which could result in our study underestimating the true prevalence of “at-risk” SOAPP®-R scores. We did not compare paper and computerized versions of the screener, which may have indicated advantages of one modality over the other.

## CONCLUSION

Our study demonstrates that it is feasible to program a tablet-based screening tool for opioid abuse potential and administer it in a time-efficient fashion to a cohort of ED patients. Patients rated the screening tool as easy to use. All enrolled patients were able to complete the tool without assistance, and required no additional staff resources for screening. The efficient completion time and patient-reported ease of completion support the conclusion that tablet computers may be used to screen ED patients.

## Figures and Tables

**Figure:**
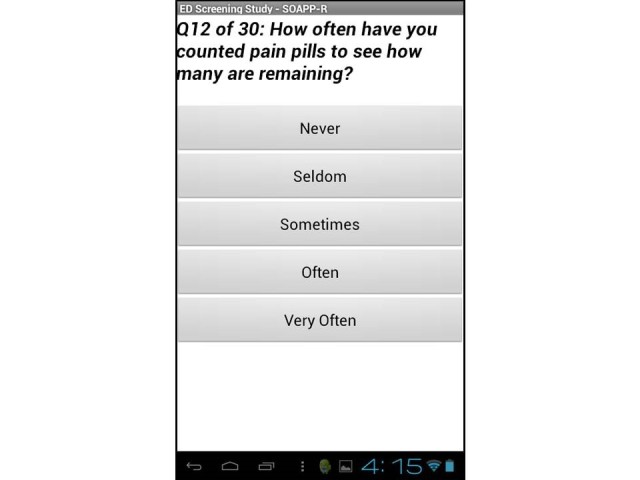
Sample screenshot of the electronic screening tool.

**Table:** Characteristics of included patients in tablet computer-based screening for possible risk for opioid abuse.

Characteristics	n (%)
Age (years)	
18–25	19 (23.2%)
26–35	16 (19.5%)
36–45	19 (23.2%)
46–55	23 (28.0%)
56-older	5 (6.1%)
Race	
White	51 (62.2%)
Black	21 (25.6%)
Asian	2 (2.4%)
Other/declined to answer	8 (9.8%)
Ethnicity	
Latino	10 (12.2%)
Not Latino	72 (87.8%)
